# Understanding the Current and Future States of Behavioural Supports Ontario: Protocol for a Mixed Methods Pilot Study

**DOI:** 10.2196/69067

**Published:** 2025-10-14

**Authors:** Jacobi Elliott, Bradley Hiebert, Danielle Domm, Karli Chalmers

**Affiliations:** 1 St. Joseph's Health Care London London, ON Canada; 2 Lawson Research Institute London, ON Canada; 3 School of Health Studies Western University London, ON Canada; 4 Arthur Labatt Family School of Nursing Western University London, ON Canada; 5 Faculty of Health Sciences Western University London, ON Canada

**Keywords:** Behavioural Supports Ontario, dementia care, responsive behaviors, family caregivers, health care providers, health care leadership, mixed methods

## Abstract

**Background:**

Behavioural Supports Ontario (BSO) was created in 2010 as a provincially defined and regionally implemented program in Ontario, Canada, to support older adults living with responsive behaviors in long-term care homes, hospitals, and at home, and to also support their family care partners. There are 14 different BSO regions in Ontario, each with its own service delivery model to provide (1) system coordination and management, (2) integrated service delivery, and (3) knowledgeable care teams and capacity building. In 2023, BSO teams across Ontario supported approximately 33,300 older adults with expressions of responsive behaviors, the majority of whom had dementia, and 20,100 family care partners.

**Objective:**

This study aimed to understand how BSO is currently affecting service providers and family care partners across all BSO care settings (ie long-term care, community, and acute care), and how BSO service delivery models can be modified to best meet the current needs of these target populations. This pilot study will be conducted in the western Ontario region, which comprises 4 different BSO regions; this will enable comparison between different BSO service delivery models to identify which service model elements may be best used to support BSO care providers, care recipients, and family care partners in the future.

**Methods:**

Following a 2-phase mixed methods sequential explanatory design, this study will invite BSO staff and leaders to complete an anonymous web-based survey, followed by focus groups and interviews to share their perspectives on BSO’s current functioning, areas of weakness, and opportunities for growth. Family care partners will also be invited to complete one-on-one interviews to share information about their experiences with BSO, areas of weakness, and opportunities for growth.

**Results:**

This study began in January 2024; data collection was completed in December 2024. Overall, 360 participants completed the anonymous survey; 51 staff and leaders and 11 family caregivers completed an interview or focus group. Data analysis is anticipated to be completed by September 2025 with results published by December 2025. Quantitative data will be analyzed using descriptive and inferential statistical techniques to identify similarities and differences in the perspectives of BSO staff and leaders across the 4 BSO regions. Qualitative data will be managed and analyzed through descriptive coding and thematic analysis to provide meaningful explanations of BSO staff, leaders, and family care partners’ experiences with the program. This study was approved by the Western University Health Sciences Research Ethics Board (124599).

**Conclusions:**

The study findings will support improvements in BSO services across the western region of Ontario. The findings will also be leveraged to support the modernization of the program to better meet the needs of patients and family care partners who depend on the service each year, while also supporting the needs of service providers within the program.

**International Registered Report Identifier (IRRID):**

DERR1-10.2196/69067

## Introduction

### Background

Behavioural Supports Ontario (BSO) is a provincially defined and regionally implemented initiative in Ontario, Canada, that is integrated across multiple care sectors and disciplines. It was created in 2010 to provide health service providers in long-term care (LTC), community-based care, and acute care with a framework to use behavioral interventions to support older adults with complex and responsive behaviors and their family care partners [1]. Expressions of responsive behaviors (ie, crying, moaning, wandering, explicit comments, sexual gestures, threats, screaming, biting, or hitting others) are often present in people living with dementia, substance use and addiction, and other neurological disorders [2,3]. Provincially, in 2023, BSO supported approximately 33,300 older adults with expressions of responsive behaviors and 20,100 family care partners across all care settings [4]. Dementia is the most common reason people receive BSO support, with 76% of residents in LTC and 80% of patients in the community having a formal dementia diagnosis [5]. In addition, patients or residents supported by BSO may also have depression, anxiety disorders, delirium, schizophrenia, and/or other psychiatric diagnoses [5].

### BSO Service Delivery Model

When BSO was created in 2010, all 14 of Ontario’s Local Health Integration Networks (ie, regional health service authorities) received funding from the Ontario Ministry of Health and Long-Term Care to establish their own regional BSO service delivery model based on local priorities and resources [6]. A single oversight group, the BSO Provincial Coordinating Office (BSO PCO), was created in 2015 to coordinate and manage the program provincially and act as an intermediary between the Ministry and the 14 BSO regional teams. This approach yielded 14 different regional service delivery models, each with its own method to provide BSO’s 3 core components: (1) system coordination and management, (2) integrated service delivery, and (3) knowledgeable care teams and capacity building [1]. Despite the Local Health Integration Networks being restructured into 6 Ontario Health (OH) regions in 2019, BSO coordination and delivery still remain within the 14 original regional models.

Regardless of the region, every BSO service delivery model is organized to include the following teams in some form [7,8]: first, a *regional lead or leads* responsible for overseeing BSO service delivery and informing resource allocation and capacity-building opportunities via specialized behavioral intervention training for all BSO staff and clinicians in their region. Second, *LTC teams* that provide behavioral support intervention care to LTC residents and support to their family care partners. LTC teams are interdisciplinary (eg, registered practical nurses, registered nurses, personal support workers, recreation therapists, behavioral therapists, social workers, and social service workers) and support one or more LTC homes, depending on the regional service delivery model. Third, *interdisciplinary multi-sector teams* that provide behavioral support services to patients and family care partners in LTC, community, or hospital. Multi-sector teams are interdisciplinary and can include registered practical nurses, registered nurses, nurse practitioners, personal support workers, recreation therapists, behavioral therapists, social workers, social service workers, occupational therapists, and physiotherapists. In some regions, these teams are managed by the regional lead, while in others, they are managed by schedule 1 hospitals, which are specially designated facilities under the Mental Health Act [9] that can admit individuals involuntarily following specific criteria and assessment from a physician.

Even though each BSO model consists of similar roles, their nature (ie, operation, management, and staff) varies greatly between regions. In addition to the above 3 teams, some regions also provide additional BSO support to patients and family care partners through other dementia-related care services, such as adult day programs or the Alzheimer Society. Despite such variation in service delivery organization, all BSO teams are united under common provincial BSO standards and common core competencies [7].

In addition, BSO service providers receive specialized education to develop the core competencies required to deliver evidence-informed behavioral health care services. Specialized education programs include standardized courses—including PIECES [10], U-First! [11], Gentle Persuasive Approaches [12], and DementiAbility [13]—and education developed by the BSO PCO. BSO health service providers extend their own learning and build capacity with other health service providers who support individuals with responsive behaviors by delivering BSO education and practical hands-on coaching of care techniques. BSO service providers also share this knowledge with family caregivers, equipping them with evidence-informed approaches to reduce the frequency and severity of responsive behaviors. The overall goal of these educational efforts is to strengthen the knowledge and skills of BSO service providers, other service providers, and family caregivers so that they can provide best-practice care to individuals with responsive behaviors.

A previous study [14] focused on the LTC Embedded Team model; however, there have been no published studies highlighting BSO’s impact on family care partners and service providers across all sectors. This pilot study will begin to address this gap by engaging with three target populations: (1) BSO service providers (ie, staff and leadership) across all BSO care settings, (2) service providers and specialists (eg, geriatricians and physicians) who are not directly funded by BSO but regularly interact with BSO staff and patients, and (3) family care partners who receive support from BSO teams to care for an older adult with complex and responsive behaviors.

This study aims to understand how BSO is currently affecting service providers and family care partners across all BSO care settings (ie, LTC, community, and acute care), and how BSO service delivery models can be modified to best meet the current needs of these target populations. Uncovering the current impacts of BSO will support the modernization of the program to better meet the needs of patients, residents, and family care partners who depend on the service each year, while also supporting the needs of service providers within the program.

## Methods

### Study Design

This study will use a 2-phase mixed methods sequential explanatory design [15,16]. In phase 1, quantitative data will be collected using an anonymous survey and analyzed by the research team. In phase 2, qualitative data will be collected using interviews and focus groups and analyzed to further explain or expand on the quantitative findings from phase 1. The sequential nature of data collection, with quantitative followed by qualitative, is well suited to provide a general understanding of an issue (the current state of BSO), while also examining nuances of the issue in more depth (family care partner and service provider experiences and areas for program growth). This design aims to use qualitative results to expand on quantitative findings and, occasionally, to understand outliers, thus enhancing interpretation to address the research problem [15,17].

**Figure 1:**
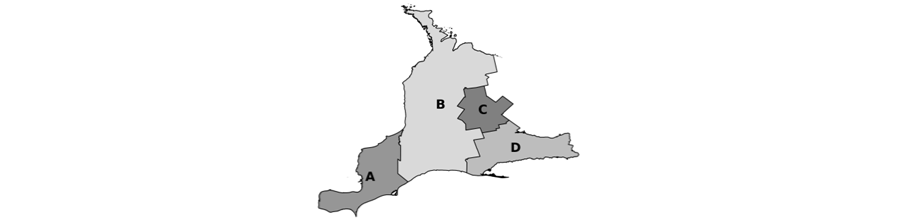
Ontario Health West regions. (A) Erie St. Clair; (B) South West; (C) Waterloo Wellington; (D) Hamilton Niagara Haldimand Brant.

This pilot study will focus on a single OH region (OH West) and its 4 BSO subregions (Erie St Clair [ESC], South West [SW], Waterloo Wellington [WW], and Hamilton Niagara Haldimand Brant [HNHB]; Figure 1). The objective of this study is to understand (1) current state of BSO across the OH West region; (2) How BSO is experienced by family care partners, staff, and clinicians across the OH West region; and (3) areas and opportunities for ongoing growth and development of the BSO program in the OH West region.

### Study Setting

This pilot study will take place in the OH West region, inclusive of its 4 BSO regions. Each BSO region operates different service delivery models (refer to Table 1 for details).

**Table 1:** Ontario Health (OH) West Behavioural Supports Ontario (BSO) region service delivery model characteristics.

Characteristic	OH West BSO region			
	Erie St Clair	South West	Waterloo Wellington	Hamilton Niagara Haldimand Brant
Lead agency	Alzheimer Society of Chatham-Kent	St Joseph’s HealthcareLondon	St Joseph’s Health Centre Guelph	Hamilton Health Sciences
Community-based care model	4 interdisciplinary multi-sector teams managed by BSO lead agency Interdisciplinary multi-sector teams provide care support to community-based adults living with dementia and their family care partners Ontario Health atHome leads community-based care to family care partners	5 interdisciplinary multi-sector teams managed by Schedule 1 hospitals in the region Interdisciplinary multi-sector teams provide direct care to community-based adults living with dementia and their family care partners Alzheimer Society provides care to community-based care partners Adult Day Programs provide overnight respite	1 interdisciplinary multi-sector team managed by lead agency Interdisciplinary multi-sector teams provide direct care to community-based adults living with dementia and their family care partners Community-based care integrated within the Specialized Geriatrics Services network Includes Community Responsive Behavior Team and BSO Transition Team	Community-based care organized by Alzheimer Society Brant Haldimand Norfolk Hamilton Halton 1 interdisciplinary multi-sector team supports community-based care for adults living with dementia and their family care partners
LTC^a^ model	Embedded teams at all 36 LTC homes in the region Each interdisciplinary multi-sector team provides advanced care support to 7-11 homes	Embedded Teams in all 75 LTC homes in region Each interdisciplinary multi-sector team provides advanced care support to 8-24 homes	Embedded Teams in all 34 LTC homes in region Interdisciplinary multi-sector team provides advanced care support to homes	5 Interdisciplinary multisector teams managed by BSO Lead Agency Interdisciplinary multi-sector teams provide direct care to LTC residents with responsive behaviors at 8-26 homes
Acute care model	Service navigator works with hospital staff at 2 major regional hospitals to support BSO care for inpatients	None	Psychogeriatric resource consultant works with hospital staff at 7 acute care sites to support BSO care for inpatients	BSO clinical leader to support BSO care for inpatients

^a^LTC: long-term care.

### Eligibility Criteria

Eligible participants across the OH West region include BSO staff and leadership at any BSO-affiliated site, including the BSO Mobile Team, LTC homes, Alzheimer Societies, adult day programs, regional lead teams, and acute care centers. Other clinicians who engage with BSO staff across the geriatric care pathway (including hospital staff, retirement home staff and leadership, family physicians, nurse practitioners, geriatric medicine specialists, geriatric psychiatry specialists, Ontario Health atHome staff and leadership, and other community-based service providers) are also eligible to participate. In addition, family care partners receiving BSO support in any care setting are eligible to participate (refer to Table 2 for the complete inclusion criteria).

**Table 2:** Participant eligibility criteria.

Participant group	Inclusion criteria
All participants	All participants must meet l of the following criteria in addition to their group-specific criteria: ability to provide consent (either signed and dated consent form, or verbal consent documented on consent form) willingness and ability to participate in study procedures can communicate in English
BSO^a^ staff and leadership	Must have completed *at least one* of the following BSO-related activities in the past 2 years: directly or indirectly provided BSO services or care to a patient participated in delivering BSO supports or services as a provider or care administrator provided care to patients who have participated in BSO programs or services
Other clinicians that engage with BSO staff	Must have completed *at least one* of the following BSO-related activities in the past 2 years: referred a patient that has previously utilized BSO services participated in delivering BSO supports or services as a health care provider or care administrator provided care to patients who have participated in BSO programs or services
Family care partners	Must meet *at least one* of the following criteria: are currently caring for or supporting a patient of the BSO program (including patients with late-stage dementia) previously cared for or supported a patient of the BSO program (including patients with late-stage dementia) within the last 2 years is currently participating as a care partner in the BSO program.

^a^BSO: Behavioural Supports Ontario.

### Sample Size

#### Phase 1: Anonymous Survey

The sample size calculation is based on data shared with the study team from each BSO regional lead team in the OH West region to estimate the total population of BSO staff and leadership (personal communication, 2024). The following criteria were used to estimate the sample size: a 10% dropout rate, power of 80%, and a statistical significance level of α=.05 [18]. This resulted in a total target sample size of 280 participants to complete the survey in phase 1 of this study.

#### Phase 2: Focus Groups and Individual Interviews

The target sample size for interviewees and focus group attendees will differ depending on the participant group, with a total target sample of up to 120 participants (up to 24 care partners and up to 96 BSO service providers or leadership) across the OH West region (Table 3).

**Table 3:** Target sample size for interviews and focus groups in phase 2.

Participant group	Targeted sample size (range)	
	Each BSO^a^ region, n	Total OH^b^ West region, n
BSO LTC^c^ home staff	2-4	8-16
BSO community-based staff	7-14	28-56
BSO leadership	2-3	8-12
Other health care providers	2-3	8-12
Family care partners	4-6	16-24
Total	17-30	68-120

^a^BSO: Behavioural Supports Ontario.

^b^OH: Ontario Health.

^c^LTC: long-term care.

### Recruitment

#### Phase 1: Anonymous Survey

A probability sampling approach will be used in phase 1 to recruit participants. Participant recruitment for phase 1 (anonymous survey) will occur through email and in-person meetings. Each BSO region’s regional lead team will distribute a study recruitment email to all BSO staff, leaders, and other eligible participants for whom they have contact information. This email will include details about the study, invite BSO service providers and leaders to participate in an optional brief survey, and inform them that they are able to participate in either phase 1 or phase 2, or both, of the study. In addition, each BSO regional lead team will provide brief details about the study and how to complete the phase 1 survey during pre-existing meetings, presentations, and other sessions. Implied consent to participate will be obtained during the initial questions of the survey.

#### Phase 2: Focus Groups and Individual Interviews

A maximum variation purposeful sampling technique [19] will be used in phase 2 to recruit participants for focus groups and individual interviews. Recruitment of BSO staff, leaders, and other health care providers who work with the BSO program will occur through email and in-person meetings. For BSO staff and leaders, details on how to volunteer for a focus group or interview will be included in the recruitment email distributed by each region’s BSO operations team, as described earlier. Staff and leaders will be able to notify the research team of their interest in taking part in a focus group or interview by entering their name, contact information, care setting (ie, LTC, community, or acute care), and whether they are staff or leadership into a contact form; the contact form can be accessed at the end of the anonymous survey in phase 1 or via a direct link. The research team will screen interested individuals and invite those who are eligible to participate in a focus group or individual interview, based on the participant’s stated preference, provided that the target sample size has not been filled for their region and participant group type. Before conducting the focus group or interview, participants will complete a brief demographic questionnaire to provide details about their background with BSO. Written consent to participate will be obtained digitally before completing the demographic questionnaire.

Family care partner recruitment for phase 2 interviews will occur in three ways: (1) email, (2) in person, or (3) a recruitment poster. A study recruitment email, including brief details about the study and information on how to participate in an interview, will be sent to BSO family care partners by either the applicable region’s BSO operations lead or an associated agency that supports BSO family care partners (eg, Alzheimer’s Society and adult day programs). Alternatively, the BSO staff will provide study information and a recruitment poster to eligible care partners. In addition, the study will be advertised by BSO staff using a visual recruitment poster with applicable study information and contact details in locations that BSO care partners frequent (eg, public locations in LTC homes and community offices). In each recruitment method, interested care partners will be required to contact the research team directly to begin study activities. Once contact is initiated, a member of the research team will explain the study in detail to the care partner and answer any questions. If the care partner is interested in participating, the researcher will walk them through the letter of information and consent form and obtain verbal consent. Before conducting the interview, care partners will complete a brief demographic questionnaire to provide details about their background with BSO.

### Data Collection

#### Overview

Data will be collected in three phases: (1) anonymous online survey, (2) focus groups and individual interviews, and (3) secondary data use. Focus groups and interviews with family care partners will occur either off-site (care partners will have the choice of an in-person interview at a local health care area) or in a Microsoft Teams meeting for a period of 9 months. Focus groups and individual interviews with BSO staff, leaders, and other health care providers will be conducted using the Microsoft Teams platform. Data will be collected by the researchers, and the principal investigators will oversee the data collection process. In phase 1, survey data will be collected; in phase 2, focus group and individual interview data will be collected; and in phase 3, secondary data from the BSO PCO will be obtained for analysis.

#### Phase 1: Anonymous Survey

BSO service providers (ie, staff and leadership) and other health care providers who support BSO from across the OH West region will be invited to participate in an anonymous online survey, hosted by Qualtrics. It is expected that it will take approximately 10 minutes to complete the survey and is accessible from any device with internet access (ie, smartphone, computer, and tablet). Survey participants will be asked demographic questions, including age, ethnicity, gender identity, professional designation (eg, nurse, personal support worker, physician, or management), sector of employment (ie, LTC, hospital, or community), the number of years practicing in their role, the number of years working with BSO, and their BSO region. Participants will also be asked a mix of scale-based and open-ended questions created by the research team to understand their perspectives on several factors related to the BSO program, including support for patients, family care partners, and staff; clarity of the BSO program in general; and relations with other BSO members and the regional lead team in their region. At the end of the survey, participants will be automatically routed to a separate form to sign up for an interview or focus group session, if they are interested. Participants will not be reimbursed or compensated for their time to complete the survey.

#### Phase 2: Focus Groups and Individual Interviews

BSO staff and leaders and BSO-adjacent health care providers who indicate their interest will be contacted by the research team to participate in an individual semistructured interview or a focus group. Semistructured interviews will take approximately 30 to 45 minutes to complete, and focus group sessions will take approximately 1 to 1.5 hours and include up to 8 participants per focus group. It is expected that 2 members of the research team will attend each interview, while 2 members of the research team will attend each focus group to facilitate the group discussion. All interviews and focus groups will be recorded and automatically transcribed using Microsoft Teams. Before the interview or focus group, participants will be asked to complete a demographic questionnaire to provide information about their age, ethnicity, gender, professional designation (eg, nurse, personal support worker, physician, or manager), sector of employment (ie, LTC, hospital, or community), the number of years practicing in their role, number of years working with or collaborating with BSO, and region. During the interview or focus group, participants will be asked about their own experiences with BSO, observations related to the impact of BSO on patients and family care partners, and suggestions for improving BSO in the future.

Family care partners who indicate their interest in the study will be invited to participate in a semistructured interview with a member of the research team, which will take approximately 30 to 45 minutes. Interviews will be conducted over Microsoft Teams, by phone, or in person, based on each participant’s preference. Following the verbal consent process and before the interview, participants will be asked to complete a demographic questionnaire to understand their age, gender, ethnicity, and caregiving situation. During the interview, participants will be asked about their experiences with BSO programs and services, observations of how BSO affected the older adult with responsive behaviors, and suggestions for improving BSO in the future. All interviews will be recorded and transcribed using Microsoft Teams.

#### Secondary Data Collection

To contextualize the findings from phases 1 and 2, the BSO PCO has agreed to provide regional administrative data for the past 5 fiscal years (FY 2019–2020 to FY 2023–2024) for the 4 OH West BSO regions. These data will provide information regarding each BSO region’s volumes of family care partners and patients and residents supported in each care sector (ie, LTC, community, and acute care), care coordination, capacity-building efforts, and system navigation support for BSO care recipients.

### Data Analysis

#### Phase 1: Anonymous Survey Analysis

Survey data will be managed and analyzed using SPSS (version 29; IBM Corp). Raw data will be screened before analyses to (1) investigate the integrity and accuracy of the data collected by identifying and removing spam and bot responses [20,21]; (2) identify, examine, and address outliers; (3) identify, examine, and address missing data; and (4) assess the statistical distribution of the data, including measures of linearity, homoscedasticity, normality, and collinearity. Cleaned data will be analyzed using descriptive and inferential statistical techniques. Descriptive statistical analyses will be conducted to create a profile of survey participants, including both personal and professional demographics, and their standing on BSO’s impact on staff, patients, and family care partners. Four-way ANOVA and factorial ANOVA will be used to identify interregional differences in continuous variables. Correlational and multiple regression analyses will be conducted to examine the relationships among the variables. Subgroup analyses will include examining differences based on sector and role type (staff or leader). Where possible, data will also be stratified based on participants’ recency of BSO interaction and the length of time spent directly or indirectly supporting the provision of BSO services.

#### Phase 2: Focus Group and Individual Interview Thematic Analysis

Qualitative analysis will be conducted using an interpretive descriptive approach [22]. Interpretive description is an inductive analytic approach used to identify and provide meaningful explanations of thematic patterns in participants’ experiences with clinical services, making it a suitable lens for analyzing the experiences of staff, leadership, and family care partners with BSO. All interview and focus group transcripts will be managed and analyzed using NVivo 14 (QSR International). At least 2 researchers will independently analyze the data to ensure reliability in coding. Data analysis will consist of line-by-line coding, followed by the identification of themes using a clustering technique [23,24]. Given the gendered nature of caregiving and health care workers, codes will be created for gender identity in our analysis [25,26], and their relationships with other themes will be examined. In addition, codes will be created for the length of time spent directly or indirectly involved with BSO services to understand the relationship between longevity and recency of a participant’s BSO interaction with other themes. If saturation is reached, that is, the point at which few or no new relevant ideas or understandings are generated from participants, no further interviews or focus groups will take place [27].

#### Secondary Data Analysis

Secondary data provided by the BSO PCO will be analyzed using descriptive and inferential statistical techniques. Descriptive statistical analyses will be conducted to describe BSO activity in the OH West region overall, and the activity of each regional BSO team (ie, ESC, SW, WW, and HNHB). Correlational and multiple regression analyses will be used to examine the relationship among variables. Repeated-measures ANOVA will be used to identify any statistically significant differences in BSO activity over time in OH West and the 4 regions.

### Ethical Considerations

Ethics approval was granted by the Western University Health Sciences Research Ethics Board (124599) and Lawson Health Research Institute Research Database Application (R-24-303). This is a nonclinical, noninterventional study and was not registered with any clinical trial registries.

### Dissemination

The study data will be published in reports and peer-reviewed journals and presented at appropriate conferences. Deidentified data will be available to all research team members. Program stakeholders responsible for sharing the findings will determine the future scaling of the program’s evaluation and quality improvement initiatives. The researchers aim to produce at least 3 peer-reviewed research manuscripts (ie, protocol, phase 1 survey results, and phase 2 interview or focus group results) for publication, to be written by the main research and implementation team.

## Results

This study was initiated in spring 2024 as part of ongoing quality improvement initiatives organized by the SW BSO regional lead team, and was supported by the BSO regional lead teams in ESC, WW, and HNHB. Data collection for the phase 1 anonymous survey concluded in October 2024; a total of 360 participants completed the anonymous survey. Data collection for the phase 2 focus groups and individual interviews concluded in December 2024, with 51 staff and leaders and 11 family caregivers completing an interview or joining a focus group. The results of the study are expected to be published in winter 2025.

## Discussion

### Anticipated Findings

Recent evidence suggests that because of the country’s aging population, the number of Canadian older adults living with dementia may increase by more than 50% by 2030 [28,29]. Such an increase would intensify the dementia-related care demands experienced by staff across all formal health care sectors [30] and family care partners of adults with dementia [28-30]. Understanding how dementia care programs, such as BSO, can be improved to meet the current needs of older adults living with dementia is a necessary step in preparing for the future care demands of Canada’s aging population.

This pilot study will provide insight into how different regional BSO service delivery models (ESC, SW, WW, and HNHB) are experienced by BSO staff and leaders, non–BSO funded health care providers who work closely with BSO teams and patients, and family care partners of older adults living with complex and responsive behaviors. Comparative analyses of how staff and leadership across the 4 regions (ESC, SW, WW, and HNHB) and care sectors (LTC, community, and acute care) experienced BSO from the phase 1 survey will be expanded upon with the phase 2 qualitative data to provide rich and nuanced insight into how BSO can be improved to better meet the needs of service providers. By including qualitative data regarding family care partners’ experiences with the program, this pilot study will also help BSO to address the current needs of the individuals it supports.

### Conclusions

This pilot study offers a first step toward understanding the current needs of BSO and areas for improvement. Collecting this information and sharing it with the appropriate BSO service providers will help prepare the program to meet the increasing dementia-related care demands anticipated during the next decade.

## Abbreviations

BSO: Behavioural Supports Ontario
ESC: Erie St Clair
HNHB: Hamilton Niagara Haldimand Brant
LTC: long-term care
OH: Ontario Health
PCO: Provincial Coordinating Office
SW: South West
WW: Waterloo Wellington
